# USING MACHINE LEARNING METHODS TO ASSESS THE RISK OF ALCOHOL MISUSE IN OLDER ADULTS

**DOI:** 10.21203/rs.3.rs-3154584/v1

**Published:** 2023-10-03

**Authors:** Matthew Wickersham, Nicholas Bartelo, Scott Kulm, Yifan Liu, Yiye Zhang, Olivier Elemento

**Affiliations:** 1. Weill-Cornell/Rockefeller/Sloan-Kettering Tri-Institutional MD-PhD Program, New York, New York, United States.; 2. Department of Physiology and Biophysics, Weill Cornell Medicine, New York, New York, United States.; 3. Department of Population Health Sciences, Weill Cornell Medicine, New York, New York, United States.; 4. Department of Emergency Medicine, Weill Cornell Medicine, New York, New York, United States.; 5. Caryl and Israel Englander Institute for Precision Medicine, Weill Cornell Medicine, New York, New York, United States.

## Abstract

The population of older adults, defined in this study as those 50 years of age or older, continues to increase every year. Substance misuse, particularly alcohol misuse, is often neglected in these individuals. To better identify older adults who might not be properly assessed for alcohol misuse, we have derived a risk assessment tool using patients from the United Kingdom Biobank (UKB), which was validated on patients in the Weill Cornell Medicine (WCM) electronic health record (EHR). The model and tooling created stratifies the risk of alcohol misuse in older adults using 10 features that are commonly found in most EHR systems. We found that the area under the receiver operating curve (AUROC) to correctly predict alcohol misuse in older adults for the UKB and WCM models were 0.84 and 0.78, respectively. We further show that of those who self-identified as having ongoing alcohol misuse in the UKB cohort, only 12.5% of these patients had any alcohol-related F.10 ICD-10 code. Extending this to the WCM cohort, we forecast that 7,838 out of 12,360 older adults with no F.10 ICD-10 code (63.4%) may be missed as having alcohol misuse in the EHR. Overall, this study importantly prioritizes the health of older adults by being able to predict alcohol misuse in an understudied population.

## Introduction

The rate of older adults, defined as 50 years of age or older, has been growing steadily over the past years. In 2010, older adults constituted 13% of the United States population, and in 2019, this has increased to 16%. Over the next 20 years, the population of older adults is estimated to be over 20% in the United States^1,2^. From a healthcare perspective, this is important, as older adults make up over 40% of hospitalizations in the United States, with over 50% of older adults having multiple chronic conditions^3,4^.

Of these chronic diseases, mental health disorders are a large proportion^5^. Studies show that over 15% of older adults suffer from mental health problems where approximately 20% of those with mental health concerns stated they had faced some sort of discrimination from their conditions^6,7^. One of these chronic diseases is late-life depression (LLD)^8^. Beyond being undiagnosed in many circumstances, LLD has less efficacious treatment options where there is a heightened recurrence following first-line antidepressant treatment compared to depression experienced earlier in life^9^. This is a major concern, as suicide rates are almost twice as high in older adults compared to the general population^10^. In addition to depression, neurocognitive disorders are highly prevalent diseases, where Alzheimer’s disease and dementia are present in 11% of older adults^11,12^.

Another mental health problem that affects older adults is substance misuse, particularly alcohol misuse^13–15^. Alcohol is the most misused substance among older adults^16^. About 50% of older adults admit to consuming alcohol, and the use of alcohol among older adult men and women has steadily increased over recent years^16^. Of these older adults, 14.5% drink more than 7 drinks per week or binge drink 5 or more alcoholic drinks on one occasion^14^. This has major health risks because the body becomes less efficient at breaking down alcohol as one gets older, leading to increased risk of comorbid diseases and alcohol poisoning^17^.

Similar to other mental health problems, alcohol misuse among older adults often goes unrecognized and untreated, often being described as an “invisible epidemic”^18^. There are various ways to screen for alcohol risk. One common method is through the Alcohol Use Disorders Identification Test (AUDIT) and AUDIT-C, a questionnaire that helps to recognize hazardous drinkers^19^. Another screening method endorsed by the American Geriatric Society suggests asking the CAGE questions (Cut down, Annoyed, Guilty, Eye-opener) to identify patients with alcohol-related problems^14^. Regarding clinical diagnoses, the Diagnostic and Statistical Manual of Mental Health Disorders, Edition 5 (DSM-V) outlines what characterizes an alcohol use disorder^20–22^. While 1.6% of older adults have been diagnosed with alcohol use disorder^23^, this number is likely underrepresented.

Although assessing alcohol risk through screening tests can be helpful, these methods have limitations^24^. An alternative approach involves applying artificial intelligence (AI) methods to predict disease and health outcomes^25,26^. In medicine, AI has already fundamentally shaped healthcare, where AI algorithms have been proven to outperform clinicians in certain circumstances^27,28^. As large health records become more accessible and abundant, AI-driven machines can synthesize and apply these vast amounts of data into helpful tools to aid in decision-making frameworks for clinical settings^29,30^.

Given the overwhelming data sources and complexities working with electronic health record (EHR) systems, processing and understanding these large datasets can be exhaustive and time-consuming. This study proposes an alternative way of approaching EHR data that involves developing a risk tool through machine learning methods on a rich dataset, namely the United Kingdom Biobank (UKB), and translating those algorithmic findings to a large EHR system. In doing so, a novel risk tool has been developed to predict and identify older adults with the highest risk for alcohol misuse.

## Methods

### Cohort selection

The United Kingdom Biobank (UKB) is a large population cohort with phenotype, genotype, and clinical information on 502,536 individuals^31,32^. Of these individuals, 146,200 are known to have never misused any substance. Conversely, 753 older adults are known to have ongoing alcohol misuse at the time of enrollment in the study. Older adults without any alcohol misuse were randomly sampled to match the size of the older adults with alcohol misuse (Supplementary Figure 1).

The Weill Cornell Medicine (WCM) EHR has a total of 2,460,461 distinct patients available. Of these patients, there are 1,745 older adults with an active diagnosis of alcohol misuse defined by having an F.10 International Classification of Diseases, Tenth Edition (ICD-10) code. Of the older adults with an F.10 diagnosis code, 643 patients had complete pack year and alcohol intake frequency information accessible in WCM’s EHR. There were 13,003 older adults without any F.10 diagnosis code who also had complete pack year and alcohol intake frequency information accessible in WCM’s EHR. Of these older adults, 643 were randomly sampled to serve as a balanced control cohort (Supplementary Figure 2).

### Machine Learning Framework and Evaluation

Classification algorithms were developed to predict older adult alcohol misuse based on clinical, demographic, and questionnaire data collected from the UKB. From this information, 103 features were selected to be used in the model. The models evaluated included logistic regression (LR), linear discriminant analysis (LDA), Gaussian Naïve Bayes (GNB), random forest (RF), eXtreme Gradient Boosting (XGBoost), multilayer perceptron (MLP), and extra tress (ET). XGBoost was chosen over traditional gradient boosting algorithms to control model complexity and prevent overfitting during the initial training.

Prior to training and testing, the data were preprocessed where categorical variables were onehot encoded, and continuous variables were normalized. Any missing values were imputed based on the mean^33^. After processing the data, the dataset was split into 80% training and 20% testing sets. The supervised learning algorithms above were applied to these sets with a 10-fold cross-validation. The success of these algorithms was determined by the area under the receiver operating curve (AUROC), the area under the precision-recall curve (AUPRC), accuracy, precision, sensitivity (recall), specificity, positive predictive value (PPV), and negative predictive value (NPV).

Feature selection was performed to identify the top variables contributing to the success of each algorithm. Using the XGBoost model, features from the UKB composite model were ranked by their importance. These top features were cross walked with those accessible in WCM’s EHR system to see what features overlap. The top 10 features that overlapped with readily available information in WCM’s EHR were chosen for the risk tool models. The risk tool model was created using the UKB cohort and assessed on the WCM cohort.

All models were developed in Python (version 3.8) using the Sci-Kit Learn^34^ and XGBoost^35^ packages (Python Software Foundation).

### SHAP Values and Risk Calculation

Shapley Additive Explanations (SHAP) values were computed for the risk calculation tooling^36^.

SHAP values help quantify the contribution of each feature to predict alcohol misuse. The risk calculation model was created by equalizing sample sizes of patients with and without alcohol misuse. Categorical variables (e.g., sex and race) were one hot encoded and normalized using minimum and maximum scaling. The data were then split into 80% training and 20% testing, where the training data were fitted using XGBoost. From the SHAP package, TreeExplainer, a method to estimate SHAP values for tree models and ensembles of trees, was used to explain the output of ensemble tree models derived from the XGBoost training set. SHAP values were then created for each patient in the testing set, and risk scores were created by summing the individual SHAP values calculated. Risk scores were normalized using minimum and maximum normalization, outputted as a range from 0 to 1. These scores were binned into five quantiles based on risk: very low, low, moderate, high, and very high.

The risk model was developed in Python (version 3.8) using SHAP (version 0.36.0) and XGBoost packages (Python Software Foundation).

### Statistical Analysis

For categorical variables, a Fischer exact test was used to test for significance of overlap. Logistic regression analysis adjusted odds ratio (OR) and 95% confidence intervals (CI) were used for age, sex, and race to identify significant associations between risk groups. Unpaired two-tailed Student’s t-tests were used to determine statistical significance between two groups. One-way analysis of variance (ANOVA) was used to assess statistical significance between three or more groups. All p-values were adjusted for multiple comparisons by Benjamini-Hochberg FDR procedure, as appropriate, and all p-values are two-sided and considered significant at the 0.05 level, unless otherwise noted.

## Results

### Demographics of Patients with and without Alcohol Misuse

The total patients in the UKB cohort (n = 1,506) and the WCM cohort (n = 1,286) were divided into two equal groups of those with and without alcohol misuse. The mean age of patients in the UKB cohort was 71.35 with a standard deviation of 4.19, and the mean age of patients in the WCM cohort was 67.67 with a standard deviation of 10.80. In both groups, there was a significant difference between those with and without alcohol misuse (p-value < 0.001) (Table 1).

In the UKB cohort, there were 418 (58.4%) females who had no alcohol misuse and 298 (41.6%) who had alcohol misuse. For the males in the UKB cohort, 335 (42.4%) had no alcohol misuse and 455 (57.6%) had alcohol misuse. In the WCM cohort, there were 349 (60.2%) females who had no alcohol misuse and 231 (39.8%) who had an alcohol misuse. For the males in the WCM cohort, 294 (41.6%) had no alcohol misuse and 412 (58.4%) had alcohol misuse. The differences between male and female for those with and without alcohol misuse were statistically significant in both the UKB cohort (OR = 1.91, 95% CI = 1.55–2.34, p-value < 0.05) and the WCM cohort (OR = 2.12, 95% CI = 1.69–2.65, p-value < 0.05) (Table 1).

Regarding race, there were no significant differences in either the UKB cohort or the WCM cohort, as assessed by a Chi-Square test. Similarly, there was no significant difference across ethnicity in the WCM cohort. Of note, there was no information available surrounding ethnicity in the UKB cohort (Table 1).

### Creating and Validating a Machine Learning Model to Predict Older Adult Alcohol Misuse

Machine learning has been used in a variety of psychiatric and substance use areas^37–39^. This study used a composite of 103 features from questionnaire, demographic, and medical data. In the composite model, XGBoost had the highest AUROC (mean = 0.88, standard deviation = 0.02) and AUPRC (mean = 0.87, standard deviation = 0.04), and LR had the highest F1-Score (mean = 0.80, standard deviation = 0.02) (Table 2). Accuracy, specificity, sensitivity, PPV and NPV were also assessed (Supplementary Table 1).

While the UKB is unique in having highly structured fields and questionnaire data, not all large hospital systems have this same information. To create a model that can be translated generally to any EHR, feature selection was performed using the XGBoost model to identify the features that were most contributive to the composite model (Supplementary Figure 3). Of the top 20 features in the composite model, only those features that are available in EHR systems were selected to be used in the model (Supplementary Table 2). The same algorithms and performance metrics were applied to the UKB cohort, with XGBoost and MLP having the highest AUROC (mean = 0.84, standard deviation = 0.02), MLP having the highest AUPRC (mean = 0.83, standard deviation = 0.02), and XGBoost having the highest F1-Score (mean = 0.79, standard deviation = 0.03) (Table 2, Supplementary Table 3).

To see how the risk tool model derived from the UKB can be applied to an EHR system, patients, and data from the WCM EHR were obtained. All the data were structured in the WCM EHR except for alcohol intake frequency and pack year. These were obtained using text extraction from the structured clinical notes. In the WCM EHR model, XGBoost had the highest AUROC (mean = 0.78, standard deviation = 0.07) and AUPRC (mean = 0.76, standard deviation = 0.05), and MLP had the highest F1-Score (mean = 0.70, standard deviation = 0.07) (Table 2). Other performance metrics were also assessed (Supplementary Table 4).

### Developing a Risk Tool to Predict Older Adult Alcohol Misuse

**Figure 1:**
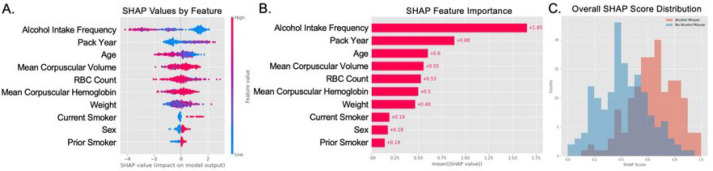
Deriving a Risk Tool Instrument to Predict Older Adult Alcohol Misuse with SHAP Scores using the UKB Cohort. (A) The distribution of SHAP values for each feature for each person. The x-axis represents the mean SHAP value for a given feature. The y-axis represents the input features. The color scheme represents the impact on the model output, with blue having a low impact and red having a high impact on the model for that specific patient. (B) Absolute SHAP importance values of the various features. The x-axis represents the mean SHAP value of a given feature. The y-axis represents the input features. (C) Comparison of SHAP values for patients with and without alcohol misuse. The x-axis is the normalized cumulative SHAP score for each patient. The y-axis is the total counts for that SHAP score. Red represents those with alcohol misuse and blue represents those without alcohol misuse.

**Figure 2:**
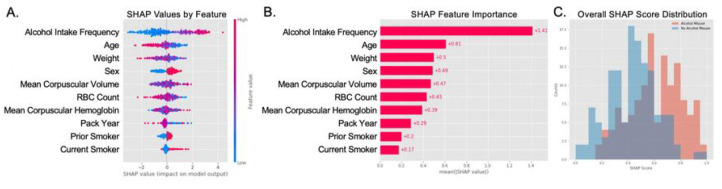
Deriving a Risk Tool Instrument to Predict Older Adult Alcohol Misuse with SHAP Scores using the WCM Cohort. (A) The distribution of SHAP values for each feature for each person. The x-axis represents the mean SHAP value for a given feature. The y-axis represents the input features. The color scheme represents the impact on the model output, with blue having a low impact and red having a high impact on the model for that specific patient. (B) Absolute SHAP importance values of the various features. The x-axis represents the mean SHAP value of a given feature. The y-axis represents the input features. (C) Comparison of SHAP values for patients with and without alcohol misuse. The x-axis is the normalized cumulative SHAP score for each patient. The y-axis is the total counts for that SHAP score. Red represents those with alcohol misuse and blue represents those without alcohol misuse.

Given XGBoost was one of the highest performing models across all performance metrics, XGBoost was selected to develop a risk tool on the UKB cohort. One way to create a risk tool is using Shapley Additive Explanations (SHAP) values^40–42^. Through this method, SHAP scores at a variable level could be negative or positive based on the inputs for a given patient (Figure 1A). The absolute SHAP feature importance revealed that alcohol intake frequency, pack year, and age were the most substantial, with a patient’s alcohol intake frequency having the overall largest impact on a total SHAP score for the cohort (Figure 1B). The distribution of all patients’ SHAP scores shows that those with alcohol misuse have an overall higher SHAP value than those without alcohol misuse (Figure 1C). To validate this risk tool, the same assessment was applied to the WCM cohort as well (Figure 2).

**Figure 3:**
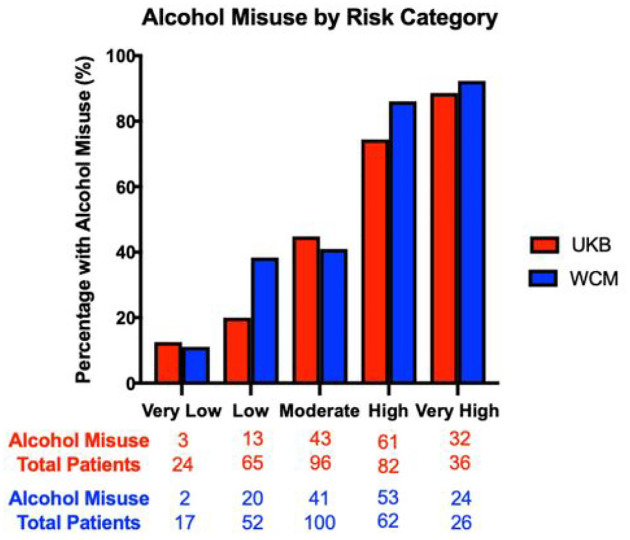
Percentage of Older Adult Alcohol Misuse by Risk Category. The x-axis indicates the ve different risk categories from the risk tool. The y-axis represents the percentage of older adults with alcohol misuse in each risk category. Red represents the UKB cohort and blue represents the WCM cohort. The counts below the bar graph represent the raw counts of those with alcohol misuse and the total number of patients that fell within that category for the UKB (red) and WCM (blue) cohorts.

To derive a risk tool based on the relative risk of a patient having alcohol misuse, different bins were created based on the normalized SHAP values. The bins created were very low risk (0.00–0.19), low risk (0.20–0.39), moderate risk (0.40–0.59), high risk (0.60–0.79), and very high risk (0.80–1.00). These bins were assessed on both the UKB and WCM cohorts, looking at the percentage of those with alcohol misuse against the total patient counts for that risk group. In both the UKB and WCM cohorts, there is an increasing percentage of alcohol misuse as the risk category becomes more severe. Of those patients in the very high-risk bin, 88.9% of patients in the UKB cohort had alcohol misuse, and 92.3% of patients in WCM cohort had alcohol misuse (Figure 3).

### Forecasting Older Adult Alcohol Misuse Using the Validated Risk Tool Instrument

**Figure 4:**
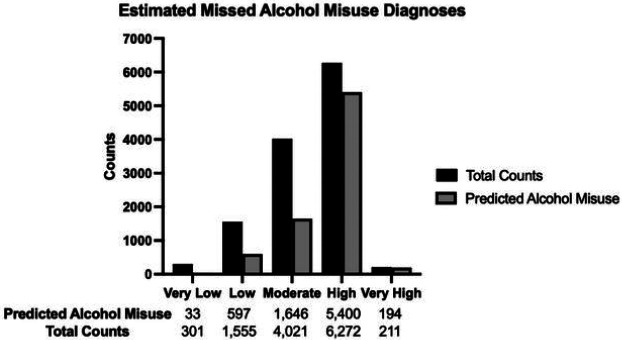
Estimated Missed Alcohol Misuse Diagnoses of Older Adults in the WCM Electronic Health Record. The x-axis indicates the ve different risk categories from the risk tool. The y-axis represents the patient counts within that category. Black represents the total number of older adults falling into that risk category, and grey represents the predicted number of patients who may have alcohol misuse, represented by an F.10 ICD-10 diagnosis code. The counts below the bar graph represent the raw values of those predicted to have alcohol misuse based on the risk tool instrument, along with the raw total counts of older adults without any F.10 ICD-10 code in the WCM EHR falling into that risk category.

In many cases, alcohol misuse in older adults is underdiagnosed with improper assessment and management^43,44^. In fact, of those who admitted to having ongoing alcohol misuse in the UKB cohort, only 12.5% of these patients had any alcohol-related F.10 ICD-10 code (Supplementary Figure 4). Given there is a large proportion of patients that could be missed having documented alcohol misuse in patient records and billing codes, the risk tool was applied to older adults without any known alcohol misuse that were not used in the validation of the initial risk tool (n = 12,360). These patients were inputted into the validated risk assessment tool, and each patient was assigned a risk category based on the patient’s calculated SHAP score. To get the predicted counts of those who have alcohol misuse but lack a proper ICD-10 diagnosis, the percentage of alcohol misuse for each respective bin in the WCM cohort was multiplied by the total counts for those SHAP values falling within that bin. For example, 11.8% (2/17) of patients binned into the very low risk category in the WCM cohort had an ICD-10 code for alcohol misuse (Figure 3). This relationship was extrapolated to our forecasting cohort, where we predicted 11.8% of the 301 patients, or 35 patients, designated as having very low risk of alcohol misuse do in fact have potential alcohol misuse based on our model. This process was repeated for each respective binning, where, overall, 7,838 patients of the total 12,360 patients (63.4%) may be missed as having any documented ICD-10 code of alcohol misuse (Figure 4).

## Discussion

The main objective of this study was to create a way to assess the risk for older adults who may be at high risk of alcohol misuse. Using the UKB repository, we developed a machine learning model of over 100 features to predict older alcohol misuse with high accuracy. Interestingly, three features related to smoking were determined to be in the top 10 most important variables from the composite model. This result is consistent with previous studies which have concluded there is a higher prevalence of smokers than non-smokers in patients misusing alcohol^45,46^.

Although the expansive model is constructive and helpful for those enrolled in the UKB program, this model cannot be readily translated to other patient populations because of the limited overlap in available data. However, through feature extraction, the top 10 important variables that were readily available across many health record systems were able to be identified to create a universal risk tool. While there was a decrease in overall performance of this risk tool compared to the composite model, the instrument still correctly identified those who are very high risk of alcohol misuse in over 90% of cases.

As noted above, there was a large discrepancy between those with ICD-10 codes for alcohol misuse and those who admitted to having ongoing alcohol addiction (Supplementary Figure 4). For the sake of comparison and the limitations of real-world data (RWD) and EHR data, alcohol misuse was defined in the WCM cohort as having any F.10 ICD-10 code. In other contexts, there may be other ways to define alcohol misuse. Namely, other cohorts could be defined by any of the following criteria: frequency of binge drinking, number of drinks per week, and/or AUDIT questionnaire scores. At the same time, the availability or combination of these different information sources can be used to enhance the risk tool to better identify those at risk of ongoing or older adult alcohol misuse. For example, AUDIT and AUDIT-C scores have performed extremely well in identifying those with alcohol misuse, including those who are older adults^47,48^. While effective, these are not continually performed on patients, and a low or high AUDIT or AUDIT-C score may not be the same longitudinally in one’s life^49,50^. Therefore, utilization of the risk tool demonstrated in this paper can lead to more frequent monitoring and assessment.

We applied our screening method to 12,360 older adults without an F.10 ICD-10 code designation of alcohol misuse. Of these 7,838 patients, 5,556 (70.9%) were predicted to be high risk or very high risk for alcohol misuse (Figure 4). This suggests a majority of older adults without a proper diagnosis are at high risk of alcohol misuse. There are a wide range of reports for alcohol misuse in older adults. Consistent with our finding, the prevalence of alcohol misuse in older adults in 2015 was estimated to be about 71% in adults aged 50–64 and about 56% in adults over 65^51^. However, other studies report a lower prevalence of alcohol misuse in this population^52^. Our risk tool is designed to be a large-scale screening tool, which may incidentally identify false positives to avoid missing older adults with alcohol misuse. Given the rates of alcohol misuse continue to increase^53^, this approach of screening may more efficiently and effectively identify individuals higher at risk to better stratify care and resources.

Despite the validation of this risk tool on an external cohort, other imitations of this study exist. Both the UKB and WCM cohorts are composed predominantly of white patients. The model performed better in the UKB cohort compared with the WCM cohort, and this could be due to the greater homogeneity of the racial makeup of this population. As larger and more diverse datasets become available, a risk with better performance for a more heterogeneous population will be possible.

Overall, this study prioritizes the health of older adults by being able to predict alcohol misuse in an exponentially growing population. As this study focused on an older population, these results should not be extrapolated to a younger group of patients. This project is novel in approach by developing a risk tool on an external dataset to be transformed and applied to larger EHR systems. In doing so, this creates new opportunities to approach EHR data and find more efficient ways to screen or flag patients who are at high risk of various health outcomes in the future.
